# ^18^ F-OC PET/CT Localization of a Phosphaturic Mesenchymal Tumor Presenting with Tumor-Induced Osteomalacia

**DOI:** 10.1055/s-0046-1823003

**Published:** 2026-05-16

**Authors:** Haifeng Hou, Lijun Zhang, Xiaofeng Zhou

**Affiliations:** 1 Department of Nuclear Medicine, Zhejiang University School of Medicine, The Second Affiliated Hospital, Hangzhou, China; 2 Department of Radiation Oncology, Zhejiang University School of Medicine, The Second Affiliated Hospital, Hangzhou, China

**Keywords:** phosphaturic mesenchymal tumor, tumor-induced osteomalacia, ^18^ F-OC PET/CT, somatostatin receptor–targeted imaging, tumor localization

## Abstract

Phosphaturic mesenchymal tumor (PMT) is an uncommon neoplasm and the leading cause of tumor-induced osteomalacia (TIO). We report a 55-year-old woman with a 2-year history of progressive hip pain and hypophosphatemia. Laboratory findings demonstrated renal phosphate wasting. Conventional imaging revealed a small soft-tissue nodule in the right thigh, and ^18^ F-AlF-NOTA-octreotide (18F-OC) PET/CT (positron emission tomography/computed tomography) showed intense radiotracer uptake, accurately localizing the culprit lesion. Surgical excision confirmed a PMT, and serum phosphate levels normalized postoperatively with resolution of symptoms. This case highlights the diagnostic value of ^18^ F-OC PET/CT in detecting small PMTs and underscores the importance of considering TIO in patients with persistent hypophosphatemia and unexplained musculoskeletal symptoms.

## Introduction

Phosphaturic mesenchymal tumor (PMT) is a rare neoplasm characterized by overproduction of fibroblast growth factor 23 (FGF-23), resulting in hypophosphatemia and tumor-induced osteomalacia (TIO). 1 Because symptoms such as chronic bone pain, muscle weakness, and recurrent fractures are nonspecific, diagnosis is often delayed. 2 Accurate tumor localization is essential, as complete surgical excision is curative in most cases. Here, we report a case of PMT localized by ^18^ F-AlF-NOTA-octreotide ( ^18^ F-OC) PET/CT (positron emission tomography/computed tomography) and confirmed pathologically, highlighting the key diagnostic considerations and imaging features.

## Case Report

A 55-year-old woman presented with recurrent pain in the left hip for more than 2 years. She had undergone a left thyroid lobectomy 4 years earlier for a thyroid nodule. She denied any history of trauma. Magnetic resonance imaging (MRI) revealed focal edema in the left femoral neck, while CT suggested an old pelvic fracture. Initial symptomatic treatment with etoricoxib (60 mg once daily) and eperisone hydrochloride (50 mg three times daily) provided only limited relief.

Six months later, the patient was re-admitted due to persistent symptoms. Laboratory evaluation showed serum creatinine 51 μmol/L, phosphate 0.58 mmol/L, calcium 2.13 mmol/L, uric acid 398 μmol/L, and parathyroid hormone (PTH) 145.92 pg/mL. Repeat testing confirmed persistently low serum phosphate (0.65 mmol/L) with elevated urinary phosphate excretion (fractional excretion 16.5%). Urine microalbumin was elevated at 46.94 mg/g. Serum and urine immunofixation electrophoresis were negative. The findings suggested renal phosphate wasting ( Table 1 ).

**Table 1:** Preoperative laboratory findings

Parameter	Value	Reference range
Serum calcium (mmol/L)	2.21	2.11–2.52
Serum phosphate (mmol/L)	0.65	0.85–1.51
Alanine aminotransferase, ALT (U/L)	323	30–120
Parathyroid hormone, PTH (pg/mL)	125.2	15.0–65.0
1,25-dihydroxyvitamin D [1,25(OH) _2_ D] (µg/L)	14.3	20.0–50.0
Urinary albumin-to-creatinine ratio (UACR, mg/g·Cr)	46.94	<25.00
Serum creatinine (µmol/L)	61.4	41.0–72.0
Estimated glomerular filtration rate, eGFR (mL/min)	98.1	>90
Serum uric acid (µmol/L)	491	154–357

**Fig. 1:**
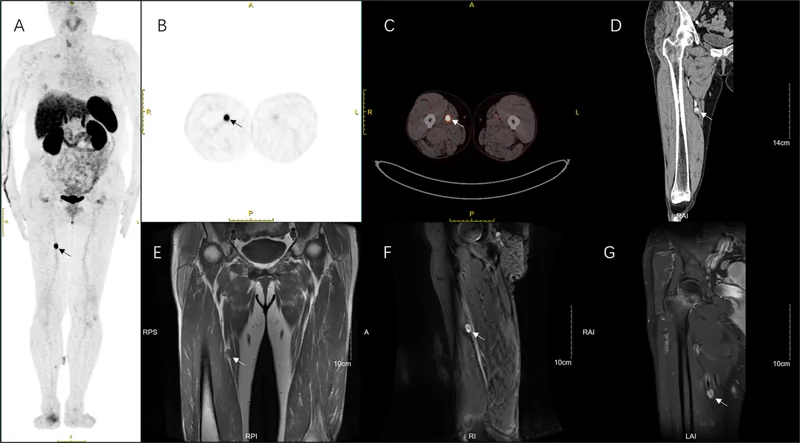
( **A** ) Maximum-intensity-projection PET image showing intense ^18^ F-OC uptake in the upper medial compartment of the right thigh. ( **B, C** ) Axial ^18^ F-OC PET and fused PET/CT images demonstrating focal tracer accumulation in the medial intermuscular compartment of the right thigh. ( **D** ) Contrast-enhanced coronal CT revealing a well-defined soft-tissue nodule (∼13 × 16 mm) adjacent to the vascular bundle in the medial mid-thigh, with marked enhancement; no abnormality was detected in the surrounding soft tissue. ( **E–G** ) MRI images showing an isointense signal on T1-weighted imaging (E, coronal view), a hyperintense signal on T2-weighted imaging (F, T2 STIR sagittal view), and avid enhancement with sharp margins on postcontrast imaging (G, T1 Flex + C coronal view). Lesions are indicated by black arrows in panels (A) and (B) and by white arrows in panels (C)–(G). CT, computed tomography; MRI, magnetic resonance imaging; PET, positron emission tomography; STIR, short tau inversion recovery.

CT confirmed a well-defined, enhancing soft-tissue nodule adjacent to the vascular bundle in the medial compartment of the right thigh (13 × 16 mm). MRI showed similar findings, with marked enhancement of the lesion and associated femoral neck marrow edema, cortical discontinuity, and a small left hip joint effusion. To further investigate the cause of hypophosphatemia, ^18^ F-OC PET/CT was performed. The scan revealed a soft-tissue nodule in the right anterior medial thigh with markedly increased uptake (SUVmax = 33.58; Fig. 1 ).

The patient subsequently underwent surgical excision. Intraoperatively, a 1.2 × 1.7 cm oval mass was identified in the medial compartment of the right thigh, adherent to the vastus medialis and adductor muscles. Histopathology revealed a spindle cell neoplasm with mild cytologic atypia and multinucleated giant cells, consistent with a PMT. Immunohistochemistry showed: CD31 (vascular +), D2–40 (focal +), CD34 (vascular +), Bcl-2 (partial +), STAT6 (−), SSTR2 (+), CD99 (partial +), CD56 (+), SATB2 (+), and K _i_ -67 (2% + ).

Postoperatively, serum phosphate and calcium gradually normalized (day 3: phosphate 0.84 mmol/L, calcium 2.06 mmol/L; day 15: phosphate 1.48 mmol/L, calcium 2.25 mmol/L). The patient's clinical symptoms resolved during follow-up.

## Discussion

PMT typically presents with TIO, manifesting as progressive bone pain, muscle weakness, and insufficiency fractures. Diagnostic delay is common, with an average interval of nearly 3 years from symptom onset to diagnosis. 3 In this case, nonspecific symptoms and unrecognized hypophosphatemia contributed to delayed identification, resulting in osteomalacia-related fractures.

Laboratory abnormalities in patients with PMT are often characteristic. Typical findings include hypophosphatemia, elevated urinary phosphate excretion, and increased alkaline phosphatase, while serum calcium generally remains within the normal range. After complete excision, phosphate levels typically normalize within 1 to 2 weeks. Although PTH is normal in most cases, this patient showed elevated PTH, likely related to decreased 1,25-dihydroxyvitamin D (1,25(OH) _2_ D) due to FGF-23-mediated suppression of renal 1-α hydroxylase. 4 In this patient, preoperative serum 1,25(OH) _2_ D was 14.3 μg/L, below the reference range of 20.0 to 50.0 μg/L, supporting this compensatory mechanism ( Table 1 ).

Histologically, PMT demonstrates variable immunoreactivity, and single markers lack specificity. 5 Recent studies suggest that SSTR2A and SATB2 co-expression may improve diagnostic accuracy. 6 In this case, positive SATB2, SSTR2, and CD56 expression were consistent with reported profiles and supported the diagnosis. SSTR2A expression also provides the biological basis for somatostatin receptor–targeted imaging. 6

PMTs are often small and difficult to detect with conventional imaging. CT and MRI may demonstrate soft-tissue masses with variable enhancement, while radiographs often reveal only bone changes secondary to osteomalacia. 7 8 Functional imaging based on somatostatin receptor targeting has significantly improved localization rates. ^68^ Ga-DOTATATE, a tracer with high affinity for SSTR2 and Food and Drug Administration-approved for neuroendocrine tumor imaging, has been used off-label for PMT localization with excellent diagnostic performance. 9 However, its widespread clinical application is limited by high cost and the short half-life of ^68^ Ga. Recently, ^18^ F-OC has emerged as a promising alternative, also demonstrating high affinity for SSTR2. Compared with ^68^ Ga-labeled somatostatin analogs, ^18^ F-OC offers several advantages, including superior spatial resolution due to the shorter positron range of ^18^ F, higher tumor-to-background contrast, and improved production scalability. 10 In the present case, whole-body ^18^ F-OC PET/CT successfully localized the culprit lesion, supporting its role as a reliable functional imaging modality for patients with TIO ( Fig. 1A–C ). Nevertheless, it should be noted that ^18^ F-OC uptake is not limited to PMTs, as other SSTR-expressing neoplasms, particularly neuroendocrine tumors, may also demonstrate increased uptake. 10

In summary, PMT should be considered in patients with chronic bone pain, recurrent fractures, and persistent hypophosphatemia. Early recognition and accurate tumor localization are essential to avoid prolonged morbidity. ^18^ F-OC PET/CT is a valuable imaging tool for detecting primary PMT lesions, facilitating definitive surgical treatment.
